# The evolutionary ecology of inbreeding depression in wild plant populations and its impact on plant mating systems

**DOI:** 10.3389/fpls.2024.1359037

**Published:** 2024-09-09

**Authors:** Pierre-Olivier Cheptou

**Affiliations:** CEFE, Univ Montpellier, CNRS, EPHE, IRD, Montpellier, France

**Keywords:** inbreeding depression, self-fertilization, natural population, population genetics, evolution

## Abstract

Inbreeding depression, the reduced fitness of inbred relative to outbred individuals was described more than two centuries ago, long before the development of population genetics. Its impact is central to evolutionary ecology and the evolution of mating systems, in particular self-fertilization in hermaphrodites. In the first half of the 20^th^ century, population genetics revealed a mechanism for inbreeding depression through homozygosity. Numerous theoretical studies have modeled inbreeding depression as a function of genetic architecture and analyzed how it varies with population selfing rates. A major concept in these models is purging, i.e., the purging of recessive deleterious mutations through inbreeding. Consequently, inbreeding depression is expected to decrease with increasing population selfing rates. Along with these theoretical studies, many experimental studies, particularly on plants, have measured inbreeding depression using experimental crosses or directly in the field. The results of these studies have revealed that the evolutionary ecology of inbreeding depression is difficult to capture and that empirical data do not exactly match model predictions, specifically purging efficacy. In addition, the lability of inbreeding depression in natural populations can qualitatively affect the selective role of inbreeding depression in the evolution of mating systems. Recently, several studies have demonstrated the role of epigenetics in shedding new light on the dynamics of inbreeding depression in natural populations. This review provides a general overview of the studies on inbreeding depression and how various angles can help capture its selective role in natural populations.

## 1 Introduction

Nearly a century before Darwin, Thomas Knight (1799) documented that vegetables with inbred plants were less fit than outbred ones, a phenomenon hereafter referred to as inbreeding depression, that is not restricted to plants; all diploid and polyploid organisms can exhibit this phenomenon. Darwin (1876) documented the deleterious effects of inbreeding in 57 species, which was described before genetics provided a mechanistic explanation. Although Gregor Mendel (1822-1884) was a contemporary of Darwin, a connection between genetics and natural selection was not established at this time. A century later, the development of population genetics provided a rationale and mechanistic model of inbreeding depression (Charlesworth and Charlesworth, 2010). Interestingly, Darwin anticipated a number of evolutionary trends, such as the relationship between inbreeding depression values and mating systems, which was later confirmed by the population genetics theory a hundred years later.

Inbreeding depression in natural populations is of central importance for several reasons. First, inbreeding depression can have consequences on population demography; this is especially true for organisms that practice regular inbreeding (e.g., self-fertilization in hermaphroditism) and this may be particularly harmful in small populations in conservation biology (Saccheri et al., 1998; Degottex-Fréy and Cheptou, 2023). Second, inbreeding depression is considered a major factor in the selection of life-history traits in organisms. The most significant trait selected by inbreeding depression is the mating system. Intuitively, a strong inbreeding depression will select outbreeding strategies to avoid the deleterious effects of inbreeding. The selective role of inbreeding depression has been studied as part of the evolution of self-fertilization in hermaphroditic organisms (e.g., Lloyd, 1979), and where organisms evolve their mating strategy through the avoidance of self-fertilization. An efficient and widespread mechanism to avoid selfing in plants is a self-incompatibility system (Nettancourt, 1977), where pollen cannot germinate on the stigma of the same plant. When inbreeding depression is not too costly, selfing can be advantageous and self-fertilization strategies are sometimes adopted by plants (e.g., Winn and Moriuchi, 2009) or animals (Ostrowski et al., 2003). Although less investigated, inbreeding depression is expected to cause evolution of dispersal traits, which is one way to avoid inbreeding depression (Ronce, 2007). Third, since the rise of population genetics, inbreeding depression has also had many connections with population and quantitative genetics, as it addresses the genetic architecture of traits. Indeed, a trait will be subject to inbreeding depression depending on the dominance of alleles coding for that trait (see Section 2), and the frequency of such alleles may change with regular inbreeding. Finally, from an empirical perspective, inbreeding depression has been estimated several times in population biology, especially in plants because plants are mostly hermaphroditic, so producing experimentally inbred and outbred progeny is easy through controlled crosses. This has led to a general formulation of inbreeding depression in hermaphrodites $\delta =\frac{W_{out}-W_{self}}{W_{out}}$ , where $W_{out}$ and $W_{self}$ capture the performance of individuals generated from outbreeding and selfing, respectively.

A long history of inbreeding depression has been reported in many outbred and inbred organisms in different environments. Such empirical data represent an unprecedented body of data that allows the testing or falsification (or at least corroborating or not) of population genetics predictions and evolutionary ecology models related to inbreeding depression.

This paper provides an overview of inbreeding depression in plants, with specific attention paid to the interplay between expectations from population genetics theory and empirical data. Far from being a simple test of theoretical models, the dialectic of theory and data has been fruitful and has enriched our understanding of the role of inbreeding depression in natural populations.

## 2 Population genetics of inbreeding depression

Inbreeding depression is defined as a reduction in the fitness for inbred progeny (e.g., selfing) compared to the fitness of outbred progeny. It is a ubiquitous phenomenon documented in various organisms such as humans, insects, birds, fish, crustaceans, ferns, and higher plants (Lynch and Walsh, 1997). Inbreeding depression results from increased homozygosity, either through crosses between related individuals or siblings or selfing, the latter of which represents the ultimate form of regular inbreeding.

### 2.1 The genetic basis of inbreeding depression

The population genetics theory of inbreeding depression is synthesized in Charlesworth and Willis (2009). Basically, the question is: what are the genetic characteristics required for fitness values to decrease because of increased homozygosity in a population? The answer can be determined by considering a single locus encoding any trait in a population and analyzing the immediate consequences of inbreeding on the fitness of that population. For simplicity, we used hermaphroditic organisms that are capable of selfing. In general, we can write a one-locus model with two alleles as:

                                                    AA          Aa        aa

Frequencies                                  D            H          R

Frequencies                                  D+H/4     H/2      R+H/4

(after one generation of selfing)

Fitness values                                *w1           w2       w3*

Frequencies before and after one generation of selfing are reported and the fitness value of each genotype can be calculated. By convention, we assume that *w1>w3>0*. The mean population fitness without selfing can be easily calculated as $\;\;\bar{w_{o}}=D\;w1+H\;w2+R\;w3$ . This can be compared with the mean population fitness after one generation of selfing as $\bar{w_{s}}=\left(D+\frac{H}{4}\right)w1+\frac{H}{2}w2+\left(R+\frac{H}{4}\right)w3$ . Inbreeding depression occurs if $\bar{w_{o}}$ > $\bar{w_{s}}$ ; i.e., $\bar{w_{o}}-\bar{w_{s}}$ > 0. One can easily show that $\bar{w_{o}}-\bar{w_{s}}=-\frac{H}{4}\left(w1-2\;w2+w3\right)$ . Thus, the sign of $\bar{w_{o}}-\bar{w_{s}}$ depends on the fitness value of the heterozygotes relative to that of the two homozygotes (w1 and w2). If alleles *A* and *a* are strictly codominant ( $w2=\frac{w1+w3}{2}$ ), there is no inbreeding depression. Therefore, strictly codominant genes do not contribute to inbreeding depression. For inbreeding depression to occur $w2>\frac{w1+w3}{2}$ ),; i.e., the dominance coefficient, *h*, of allele *a* over *A* is below 0.5. Indeed, the condition can be rewritten as $w2=\frac{w1+h.w3}{2},\;h<0.5$ . The one-locus rationale can be extended to multi-locus traits, with two genetic hypotheses fulfilling the condition that alleles contribute to inbreeding depression (Charlesworth and Willis, 2009). The first is the partial dominance hypothesis (0<h<1/2), where partially recessive deleterious alleles arise owing to mutations; and the second is the overdominance hypothesis, where heterozygotes are fitter than homozygotes (h<0). The relative contributions of over- and partial-dominance were subject to intensive debate in the 1970s (Crow, 1993). However, it is now accepted that the partial dominance hypothesis is a major cause of inbreeding depression (Charlesworth and Willis, 2009; Hedrick and Garcia-Dorado, 2016). Empirical studies that measured mutation parameters have concluded that the rate of new deleterious mutations lies in the range of 0.1 to 1.0 per zygote per generation, with the reduction in fitness between 1% and 10% in the homozygous state in metazoans (Schoen, 2005). The implication of the population genetics theory of inbreeding depression is that it is not static and depends on the genetic architecture of the traits (e.g., mutation and dominance) and selection, which lowers the frequency of deleterious alleles. Deleterious alleles are expected to be maintained at mutation/selection equilibrium in large populations, eventually subject to genetic drift when population sizes are small. Experiments measuring plant fitness can be used to make inferences about the deleterious effects of mutations but do not identify specific loci that generate inbreeding depression. Genetic mapping studies are now possible due to the development of intensive gene markers. Quantitative trait locus (QTL) mapping can identify the specific loci that cause inbreeding depression and such an approach has been investigated mostly in crop plants such as maize (Charlesworth and Willis, 2009).

### 2.2 Purging inbreeding depression or how inbreeding depression changes with population inbreeding

The genetic basis of inbreeding depression implies that it can change with regular inbreeding in populations. When deleterious recessive mutations cause inbreeding depression, the magnitude of inbreeding depression (δ) depends on the frequency of the deleterious mutations. While such a frequency is expected to be $q=\frac{\mu }{h.s}\;(\text{for h}\neq 0$ ) in a fully outcrossing populations (i.e., random mating), where μ, h, and s are the mutation rate, the dominance coefficient, and deleterious effect of the recessive homozygotes, respectively, the frequency is $\frac{\mu }{s}$ for a fully selfing population and $\frac{\mu }{s}<\frac{\mu }{h.s}$ for *h<*1 (Charlesworth and Charlesworth, 2010). Under the partial dominance hypothesis, the purging process is defined as a reduced frequency of deleterious mutations in inbred populations. While deleterious mutations can be maintained in the heterozygous state in outcrossing populations because they have little effect on fitness, inbred populations are expected to eliminate most of their deleterious mutations because of the higher homozygosity in that population. The concept of purging has been central to the population genetics of inbreeding depression and predicts that outcrossing populations should exhibit stronger inbreeding depression than selfing populations. Multilocus models have been developed to predict the amount of inbreeding depression over the whole genome as a function of the population selfing rate (Charlesworth et al., 1990; Charlesworth and Willis, 2009). Although partial dominance can account for inbreeding depression in wild populations, it is important to note that in the overdominance hypothesis, inbreeding depression is expected to increase with population selfing rates.

## 3 Empirical estimates of inbreeding depression in plants

### 3.1 The phenotypic expression of inbreeding depression in plants

Hermaphroditic plants are ideal candidates for estimating inbreeding depression because experimental crosses (e.g., hand pollination) easily generate selfed progenies (except in SI species) and outcrossed progenies from the same mother plant. Measuring progeny fitness from experimental crosses allows for parameter estimation. Inbreeding depression is expected to express throughout a plant’s life. In annual plants, it is classically estimated at four stages of the life cycle: seed set, germination, survival before reproduction, and final biomass or number of flowers as a proxy for progeny number (Husband and Schemske, 1996). **Table 1** shows the mean magnitudes of inbreeding depression in angiosperms, gymnosperms, and gynodioecious species (Data reproduced from Winn et al., 2011). Using stage values, the inbreeding depression was expressed over the entire plant cycle; however, a limitation of these datasets is that only annual species were studied. In perennials, an additional stage must be considered to calculate the survival rate of the next generation. Although this parameter is often difficult to estimate directly because of the duration of the experiments, there is evidence that the survival stages may be subject to inbreeding depression (Delmas et al., 2014), in accordance with the fact that perennials are mostly outcrossers (Morgan et al., 1997).

**Table 1:** Means ( ± SE) inbreeding depression expressed at the four successive life‐cycle stages: seed set, germination, survival before reproduction, growth/reproduction for all taxa, angiosperms only, gymnosperms only, and gynodioecious taxa only.

	N	Seed set	Germination	Survival to flowering	Growth/reproduction
All taxa	68	0.206 (0.032)	0.116 (0.018)	0.134 (0.032)	0.220 (0.019)
Angiosperms	58	0.143 (0.026)	0.127 (0.019)	0.119 (0.035)	0.226 (0.024)
Gymnosperms	10	0.571 (0.098)	0.053 (0.032)	0.211 (0.089)	0.187 (0.033)
Gynodioecious taxa	10	0.287 (0.073)	0.239 (0.051)	0.234 (0.149)	0.247 (0.065)

Data taken from Winn et al. (2011).

### 3.2 Does inbreeding depression decrease with population selfing rates?

**Figure 1:**
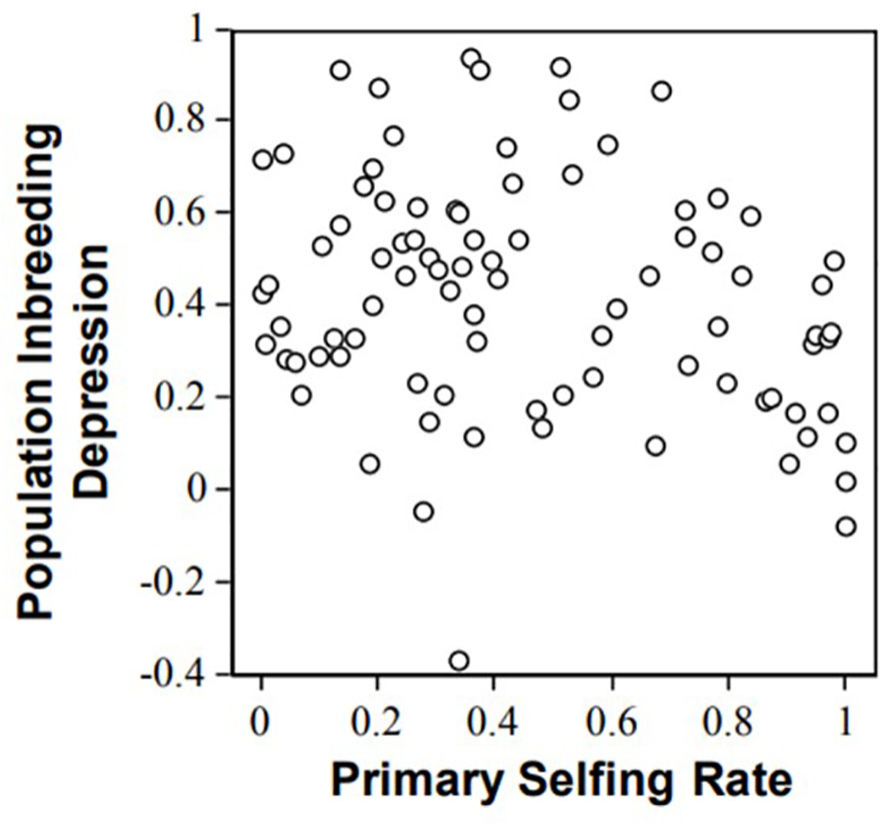
Relationships between inbreeding depression and population selfing rates illustrated with progeny array analysis using microsatellites in 87 plant populations (from Winn et al., 2011).

In plants, not only can inbreeding depression be experimentally estimated, but the selfing rate from the studied populations under natural conditions can also be precisely estimated due to neutral co-dominant markers (e.g., microsatellites, Jarne and Lagoda, 1996). The relationship between inbreeding depression over the entire life cycle and population selfing rate illustrates the possibility of purging. Data collect for 87 species (angiosperms and gymnosperms; (Winn et al., 2011) show a general trend of inbreeding depression decrease with selfing (**Figure 1**). However, the correlation between inbreeding depression and selfing rate was weak (Spearmann rank correlation, rho = −0.18, one-tailed P = 0.1, Winn et al., 2011); that is, selfing explains only a fraction of the variance in inbreeding depression estimates (**Figure 1**). This questions the ability of the population genetics model to predict inbreeding depression values. For instance, the genus *Amsinckia* has low and nearly similar inbreeding depression values in a set of species despite their contrasting mating systems (Johnston and Schoen, 1996). Such discrepancies between the models and data led (Byers and Waller, 1999) to tone down the importance of purging dynamics in inbreeding depression. More recently, Toczydlowski and Waller (2023) analyzed 12 populations of *Impatiens capensis* spanning a broad range of individual (-0.17 to 0.98) and population (F = 0.25 - 0.87) inbreeding. Unexpectedly, they concluded that inbreeding depression was not systematically lower in inbred populations.

Several arguments have been proposed to account for the differences between model expectations and experimental data. First, genetic drift can lower the efficacy of selection against deleterious mutations, especially when the mutation effects are small, which can blur the relationship between selfing rate and inbreeding depression Winn et al. (2011) suggested that selective interference could prevent purging and explained the weak relationship between inbreeding depression and selfing observed in empirical studies. These hypotheses further suggest that the dynamics of inbreeding depression are not fully captured by the deleterious mutation model. In addition Winn et al. (2011) reported an intriguing trend for mixed selfers (0.2< s< 0.8) that exhibited higher inbreeding depression than selfers (s>0.8) and outcrossers (s<0.2). However, this trend must be interpreted with caution because it could be an artifact of noise within the experimental crosses. Experimental crosses are often performed in insect-proof greenhouses and outcrossers often produce a large amount of pollen grains, so there is the possibility that the supposedly “selfing” class of crosses to be polluted by outcrossing events. Such pollen pollution would result in decreased contrast between the “outcrossing” and the “selfing” classes of plants, and explain the observed trend. Because mixed selfers produce far less pollen, this bias was not expected (or at least less expected) in mixed selfers. Pollen pollution can be controlled by genotyping the progeny, which advocates the use of genetic markers for accurate inbreeding depression estimates.

While most of our theoretical knowledge is based on diploids, inbreeding depression has been estimated in polyploidy species. Clo and Kolar (2022) reported that phylogenetically young polyploid lineages have a lower amount of inbreeding depression than their diploid relatives. The authors suggest the negative effect of polyploidy on the magnitude of inbreeding depression tends to decrease with time since polyploidization. In crops, Li and Brummer (2009) suggest that the higher inbreeding depression observed in some tetraploids could result from the rapid loss of multiple-allelic interactions within a locus.

### 3.3 Field estimates of inbreeding depression and the ecology of inbreeding depression

In Darwin’s book The Effect of Cross- and Self-Fertilization in the Vegetable Kingdom, he wrote:

*The result was in several cases (but not so invariably as might have been expected) that the crossed plants did not exceed in height the self-fertilized in nearly so great a degree as when grown in pairs in the pots. Thus, with the plants Digitalis, which competed together in pots, the crossed were to the self-fertilized in height as 100 to 70; whilst those which were grown separately were only as 100 to 85. Nearly the same result was observed with Brassica. With Nicotiana the crossed were to the self-fertilized in height, when grown extremely crowded together in pots, as 100 to 54; when grown much less crowded in pots as 100 to 66, and when grown in the open ground, so as to be subjected to but little competition, as 100 to 72 (Darwin, 1876)*.

If Darwin’s hypothesis was that inbreeding depression is likely lower in inbred populations so that selfing populations pay a low cost of self-fertilization, he also noted that environmental conditions may affect inbreeding depression values. This hypothesis was not explored until the 1980s, most of the studies were performed in a single constant environment (greenhouses, common gardens). Inbreeding has been found to compromise host plant defense gene expression in *Solanum carolinense* (Campbell et al., 2013). In the 2000’s, several studies have included the effect of environmental conditions on inbreeding depression estimates (reviewed in Crnokrak and Roff, 1999; Armbruster and Reed, 2005; Cheptou and Donohue, 2011). However, the results show contrasting trends. Armbruster and Reed (2005) concluded that stress does not necessarily increase inbreeding depression, while Cheptou and Donohue (2011) reported that both higher or lower inbreeding depression could potentially be found. By modelling inbreeding depression in a quantitative genetic model, Ronce et al. (2009) found that inbreeding depression would decrease under stressful conditions. The contrasting results may be due to that fact that stress applied in experiments encompasses a variety of stress that may act differently on genotypic expression.

The question is whether inbreeding depression measured under standard conditions provides a relevant estimate of inbreeding depression in natural populations and Dole and Ritland (1993) made interesting contributions in this regard. They estimated the inbreeding depression directly in the field in two species of the *Mimulus* genus over two years. These inferences were based on changes in population inbreeding from seeds to adults (**Table 2**). Interestingly, they found that the inbreeding depression estimates were approximately three times stronger in 1989 than 1990 for both taxa. Because changes in deleterious gene frequency (i.e., purging) could not account for such inbreeding depression variations in a single generation, they concluded that inbreeding depression is either affected by environmental conditions in natural environments or that inbreeding depression likely varies from year to year from the stochasticity of environmental conditions.

**Table 2:** Field estimates of inbeeding depression for two consecutive years in the genus *Mimulus*.

	Relative fitness of selfed progeny	
	*Mimulus guttatus*	*Mimulus platycalix*
1989	0.09	0.17
1990	0.28	0.49

Data from (Dole and Ritland, 1993).

The fact that environmental factors are likely to affect inbreeding depression suggests that recessive mutations are not unconditionally deleterious, but that their selective effect varies with the environment. It is also possible that certain alleles are beneficial in certain environments and deleterious in others. For instance, in the species *Silene latofolia* Schrieber et al. (2019) reported that the magnitude of inbreeding depression in fruit number was lower in invasive than native populations. While we have compelling evidence of inbreeding x environment interactions on fitness, the ecology of inbreeding depression remains incomplete and we are yet to identify the relevant factors affecting the magnitude of inbreeding depression. Some ecological factors may be inherent to the population, such as density dependence, whereas others may be caused by external factors like climate and interspecific competition. Regarding density dependence, Cheptou and Schoen (2003) estimated inbreeding depression in selfing and outcrossing *Amsinckia* species (Johnston and Schoen, 1996), which included competition at high density and between inbred and outbred plants. The rationale was that, in a selfing population, the most competitive interactions occur within the inbred plants, whereas in an outcrossing population, the most competitive interactions occur with outbred plants. Interestingly, a larger inbreeding depression was found in outcrosser taxa than in selfing taxa, which was consistent with the evolutionary models of selfing.

## 4 Demographic consequences of inbreeding depression in natural populations

Owing to its effects on fitness, inbreeding depression is expected to affect population demography, which is a concern in conservation biology (Frankham, 1995). Saccheri et al. (1998) provided empirical data that demonstrated that inbreeding can affect population extinction. In the plant species *Gentiannella campestris*, Lennartsson (2002) reported on the role of self-fertilization in population persistence in the field, showing that selfers suffer from a demographic disadvantage compared with outcrossers and that the time to extinction was greatly reduced in selfers when compared to that of outcrossers. This was attributed to inbreeding depression over the entire life cycle of the plant; however, this result was not consistent with population genetics predictions. Indeed, at equilibrium, the genetic load of a self-fertilizing species is expected to be equal to the mutation rate µ, or half the genetic load of an outcrossing population, 2 µ (Charlesworth and Charlesworth, 2010). The discrepancy between the empirical data and models suggests that other forces can affect inbreeding depression. For example, it is possible that populations have not reached equilibrium, especially when endangered species may be out of their evolutionary equilibrium.

## 5 Evolutionary consequences of inbreeding depression in natural populations

In wild populations, we classically consider dispersal and mating to be two major traits influenced by inbreeding depression. Among the different causes of evolution, dispersal has been identified as a mechanism to avoid the deleterious effects of inbreeding when populations are genetically structured; i.e., when individuals within the population are genetically related (Bengtsson, 1978). However, inbreeding avoidance has not been considered a major factor in the evolution of plant dispersal, rather, inbreeding avoidance is thought to play a role in animals such as mammals, favoring male-biased dispersal. In contrast, plant mating system evolution has been closely linked to inbreeding depression since Lloyd’s seminal work (Lloyd, 1979). In these models, outcrossing avoids inbreeding depression, while selfing is counter-selected by inbreeding depression, providing a gene transmission advantage over outcrossing genes (Fisher, 1941) and an ecological advantage through reproductive assurance. Mathematically, the evolution of self-fertilization can be captured by analyzing the fate of a rare selfing mutant s in a population with the mean selfing rate $\bar{s}$ . The fitness of the rare mutant can be captured by summing the genes transmitted to the next generation through selfed seeds, outcrossed seeds, and pollen export in the population:


(1) $$\begin{array}{l} \text{Selfed seeds Outcrossed seeds Pollen export}\\ w\left(s,\bar{s}\right)=f\left[2.s\;\left(1-\delta \right)\;+\;\;\;\;\left(1-s\right)\;\;\;\;\;\;\;\;\;+\;\;\;\;\;(1-\bar{s)}\right] \end{array}$$

where δ is the inbreeding depression parameter and *f* is the number of ovules produced by an individual. It is important to note that the selfed seeds contain two copies of the parental genes, whereas the outcrossed seeds contained only one copy. From this classical model (Lande and Schemske, 1985), it follows that inbreeding depression is the only parameter that influences the evolution of self-fertilization. If the inbreeding depression δ > 0.5, outcrossing is always favored and 100% outcrossing will evolve in the population. In contrast, if inbreeding depression δ< 0.5, selfing is always favored and 100% selfing will evolve in the population. This simplistic model has been criticized because it assumes that inbreeding depression is constant; i.e., it does not account for purging process (see part 1). Population genetic models have been developed to account for the joint evolution of selfing and inbreeding depressions (Lande and Schemske, 1985; Charlesworth et al., 1990), in particular the importance of association between inbreeding depression loci and selfing modifiers loci (Uyenoyama et al., 1993). Interestingly, the qualitative behavior of the model predictions did not change; either 100% selfing or 100% outcrossing evolved in the population depending on the initial inbreeding depression value. In these models, purging acts as positive feedback on selfing because purging decreases inbreeding depression, favoring the evolution of selfing. Many theoretical models have been developed to analyze the evolution of self-fertilization, in particular to explain stable mixed mating systems (Goodwillie et al., 2005). Here, I will focus on a specific mechanisms maintaining stable mixed selfing rates, based on environment-depedant inbreeding depression.

In 2001, Cheptou and Mathias (2001) proposed that, in natural populations, the magnitude of inbreeding depression may be subject to fluctuations in time or space due to environmental variations (e.g., abiotic or biotic factors). They demonstrated that qualitative changes in the evolution of selfing occur because of inbreeding depression variation; in particular, mixed selfing rates are stabilized. First, they found that temporal but not spatial variation could maintain mixed selfing. Second, they showed that spatiotemporal variation may evolve into polymorphisms in selfing rates. The temporal variation case can be illustrated using Lloyd’s model. From Equation 1, we can rewrite the fitness of the rare mutant, assuming that inbreeding depression changes over time:


(2) $$wt\left(s,\bar{s}\right)=f\left[2.s\;\left(1-\delta \left(t\right)\right)\;+\;\left(1-s\right)\;+\;(1-\bar{s)}\right]$$

We assume that δ varies in time in a stochastic manner where in each generation, δ € [0,1], is a probability distribution. In this context, the relevant fitness measure is the geometric mean over time.


$$W=\underset{n\to \infty }{\lim}{\left(\prod_{t=1}^{n}wt\right)}^{1/n}$$

**Figure 2:**
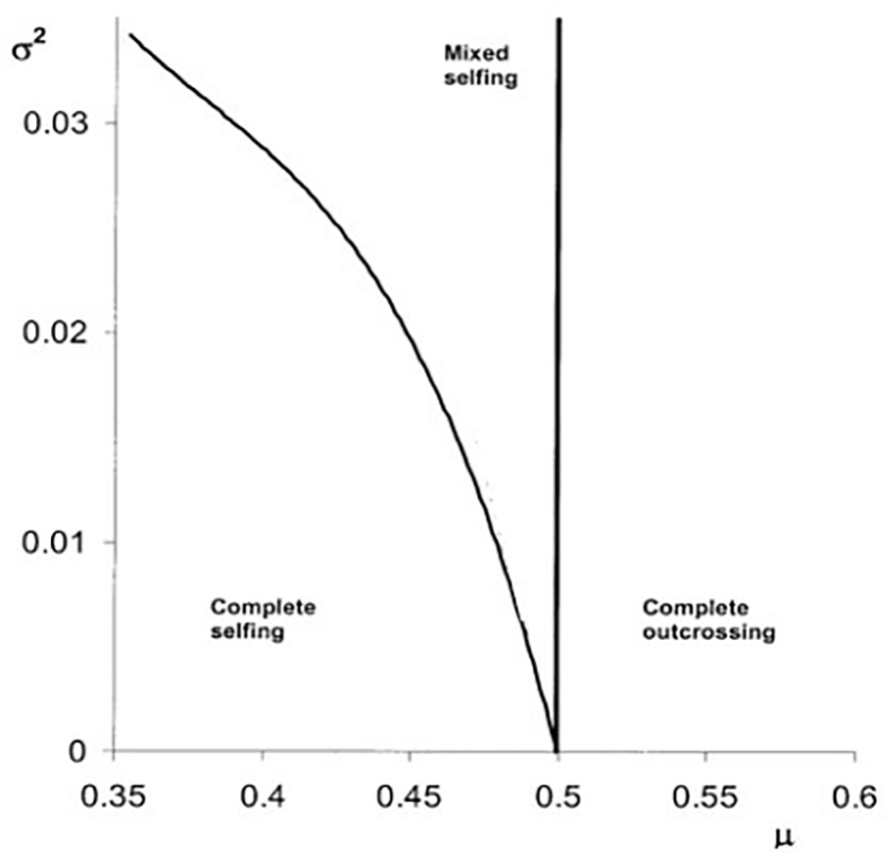
The evolution of self-fertilization based on Equation 2 (see main text) under temporal variation of inbreeding depression. The different evolutionary stable strategies (e.g., complete selfing, mixed selfing, and complete outcrossing) are depicted as a function of the mean (μ) and the temporal variance (σ²) of the inbreeding depression distribution using a truncated Gaussian distribution (0<δ<1). Boundaries among complete selfing and mixed selfing and complete outcrossing are depicted by the black lines (based on Cheptou and Schoen, 2002).

**Figure 2** depicts the selfing rate evolution, assuming a truncated Gaussian distribution (δ ϵ [0,1]) (Cheptou and Schoen, 2002). If the temporal variance is higher than zero, there is a possibility that mixed selfing rates will evolve.

Overall, the evolutionary models predicted that high inbreeding depression counteracts selfing strategies; however, the empirical data do not always follow this approach. Delmas et al. (2014) reported interesting results from studying the perennial mass-flowering shrub *Rhododendron ferrugineum*. They compared the parental inbreeding coefficient with the progeny selfing rate for each parent and in absence of inbreeding depression, it was expected that at equilibrium, $F_{parent}=\frac{s}{2-s}$ , where *s* is the selfing rate in the progeny (Charlesworth and Charlesworth, 2010). This study revealed that despite the high selfing rate in the progeny, $F_{parent}$ was close to zero, which requires very high inbreeding depression values (δ>0.9) despite the high selfing rates caused by the absence of pollination agents. Such high inbreeding depression values are inconsistent with expectations for selfing species. A possible explanation for this outcome is that the population has not reached equilibrium because of the recent decline in pollination, and/or inbreeding depression remains strong in perennial species and the long generation time does not easily allow for the purging deleterious mutations (see also Scofield and Schultz, 2006).

We have learned much about the dynamics of inbreeding depression by comparing the model expectations and empirical estimates of inbreeding depression. However, there are discrepancies between predictions and data.

## 6 Revisiting inbreeding depression through the lens of epigenetics

**Figure 3:**
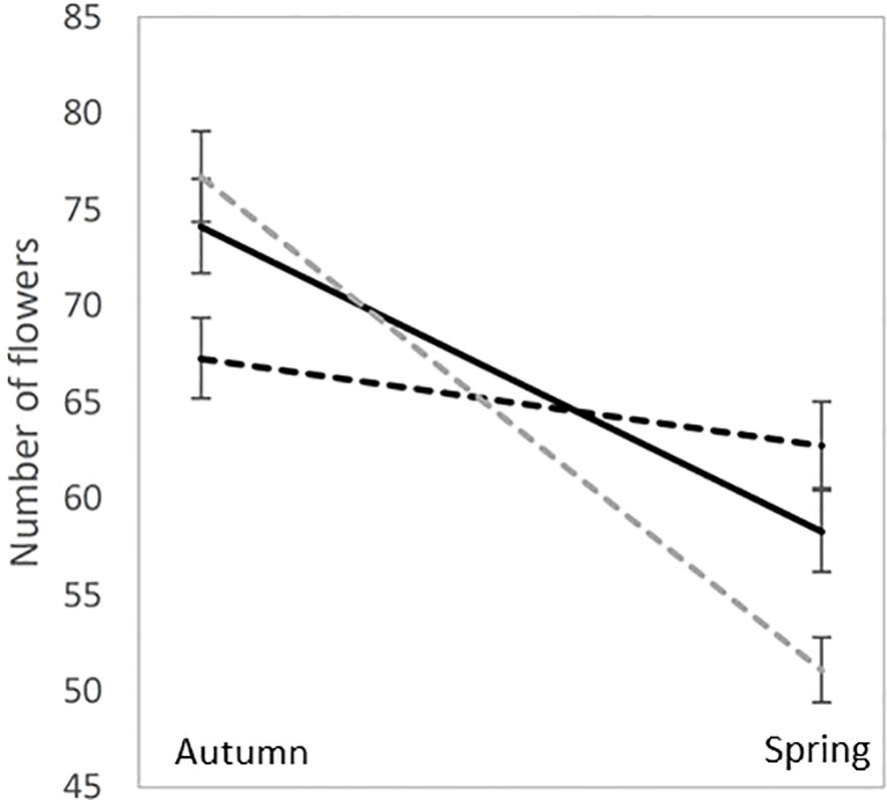
Mean and standard errors for the number of flowers in *Lamium amplexicaule* experimental crosses: outcrossed progeny (solid black line), Chasmogamous selfed progeny (dotted black lines) and Cleistogamous selfed progeny (dotted gray lines). The experiment was performed in an experimental garden both in the spring and in autumn, which are the two flowering seasons for *L. amplexicaule* natural populations.

Until recently, most of our understanding of inbreeding depression in natural populations has resulted from the interpretation of inbreeding depression estimates in common environments using population genetics and, more specifically, the dynamics of deleterious mutations in natural populations. Recently, several studies have explored the potential role of epigenetics in the magnitude of inbreeding depression. While the epigenetics of inbreeding depression are still in their infancy, these results can change our understanding of inbreeding depression in natural populations and are worth discussing. *Sensu lato* epigenetics can be defined as any biological factor that affects phenotypes without altering the DNA sequence. In evolutionary ecology, methylation is one of several types of epigenetic marks that regulate DNA expression. Stojanova et al. (2020) measured inbreeding depression in the species *Lamium amplexicaule*, which produces cleistogamous and chasmogamous flowers in various proportions. They produced inbred progeny by hand self-pollination in chasmogamous and cleistogamous flowers (i.e., obligatory selfed), as well as produced outbred progeny from hand-outcrossed pollinations. According to the classical inbreeding depression theory, it was expected that progeny from cleistogamous flowers to behave like self-pollinated progeny from chasmogamous flowers, having a lower fitness than outbred progeny from chasmogamous flowers. The striking result of the study was that the “flower effect” on progeny fitness was statistically significant and had a higher impact on fitness than the “inbred/outbred” status of progeny, in spite of no major difference in seed sizes. Because *L. amplexicaule* flowers both in spring and autumn, with a higher cleistogamy rate in autumn, the authors tested the influence of season on progeny fitness and discovered that cleistogamous progeny performed better in autumn than in spring, while the reverse was true for both inbred and outbred chasmogamous progeny (**Figure 3**). However, this fitness pattern was not consistent with adaptation to environment-dependent inbreeding depression but was possibly consistent with adaptation to seasonal pollinator activity. Also, in *Solanum corolinense* Nihranz et al. (2020) found transgenerational effects of herbivory and maternal plant inbreeding. In particular, they found that offspring of damaged plants flowered earlier and produced more flowers than offspring of undamaged plants.

This result is puzzling and suggests that epigenetic mechanisms are involved in the magnitude of inbreeding depression beyond the DNA sequence. Vergeer et al. (2012) conducted a simple and inspiring experiment on the role of methylation in inbreeding depression magnitude. The authors performed a classical inbreeding depression experiment by comparing selfed and outcrossed offspring in *Scabiosa columbaria* and concluded that inbreeding depression was high in this species and that inbred individuals had higher levels of methylation than outbred individuals. Then, they applied a chemical demethylation treatment (5-azacytidine) to the seedlings and found that the inbreeding depression was nearly zero in the treated plants (and inbred plants after treatment were taller than without treatment), clearly demonstrating that methylation is involved in inbreeding depression (Biémont, 2010; Han et al., 2021). However, the way methylation group act in interaction with inbreeding on fitness is not clearcut. In their review, Nebert et al. (2010) concluded that epigenetics on inbreeding depression can be either beneficial or detrimental.

Several lessons were learned from these experiments. First, they provide mechanisms for environment-dependent inbreeding depression. Epigenetic marks can be generated by environmental conditions (e.g., stress), which can affect the magnitude of inbreeding depression. Second, as noted by Han et al. (2021), the magnitude of inbreeding depression cannot be fully captured by the dynamics of “unconditional” deleterious mutations in populations. The experiments discussed suggest that inbreeding depression may be evolutionarily labile and that it includes epigenetic regulation. This opens a new perspective in inbreeding depression studies, in which ecological factors must be included to understand the dynamics of inbreeding depression in natural populations and its evolutionary consequences on life history traits. In the future, analyzing patterns of epigenetics (e.g. methylation) in relation to ecological factors should help to clarify how epigenetics modulate inbreeding depression and if such epigenetic patterns are adaptive with regards to natural selection in the wild.

## 7 Conclusion

More than two centuries after its discovery, inbreeding depression has remained an active area of research. Inbreeding depression in plants has been closely linked to the evolution of plant mating systems for two reasons. First, inbreeding depression values are expected to counter-select selfing genes, and evolutionary models have characterized inbreeding depression values that prevent selfing genes from evolving in the population (see the δ-threshold, part 4, Lloyd, 1979). Secondly, population genetics has established that the extent of inbreeding depression is linked to selfing rates (i.e., the purging process). However, these two approaches are distinct and even after 40 years of research, the link between inbreeding depression and selfing remains unclear (Byers and Waller, 1999).

Although much progress has been made in population genetics to capture the genomic architecture of inbreeding depression, more recent studies have revealed that the magnitude of inbreeding depression cannot rule out the importance of environmental conditions. This is especially important when analyzing the selective role of inbreeding depression in mating systems. Indeed, the value of inbreeding depression in favoring selfing genes is meaningful. The epigenetic approach *sensu lato* appears to be promising for investigating inbreeding depression in natural populations (Han et al., 2021) and is likely to enrich our understanding of the genetics underlying inbreeding depression.
